# Perfil de nutrientes de productos alimentarios eximidos de la aplicación de advertencias en el frente del envase durante la primera etapa de la Ley de alimentación saludable en Perú: estudio de caso

**DOI:** 10.26633/RPSP.2021.153

**Published:** 2021-12-16

**Authors:** Jaime Delgado Zegarra, Fabio da Silva Gomes

**Affiliations:** 1 Instituto de Consumo de la Universidad de San Martín de Porres Instituto de Consumo de la Universidad de San Martín de Porres Lima Perú; 2 Organización Panamericana de la Salud Organización Panamericana de la Salud Washington D.C. Estados Unidos de América

**Keywords:** Etiquetado de alimentos, nutrición, alimentación y dieta, legislación alimentaria, obesidad, programas y políticas de nutrición y alimentación, Perú, Food labeling, diet, food, and nutrition, legislation, food, obesity, nutrition programs and policies, Peru, Rotulagem de alimentos, alimentos, dieta e nutrição, legislação sobre alimentos, obesidade, programas e políticas de nutrição e alimentação, Peru

## Abstract

**Objetivo.:**

Identificar el perfil nutricional de productos alimentarios eximidos de presentar uno o más sellos de advertencia nutricional en el frente del envase durante la primera etapa de vigencia de la Ley 30021 de Promoción de la Alimentación Saludable para Niños, Niñas y Adolescentes de Perú.

**Métodos.:**

Se recolectaron datos de 188 productos desde puntos de venta de la ciudad de Lima. La muestra por conveniencia incluyó productos que hasta el 17 de septiembre de 2021 estaban eximidos por la legislación de presentar alguna de las advertencias para el azúcar, el sodio o las grasas saturadas. Se evaluó la proporción de productos que estarían obligados a aplicar uno o más sellos de advertencia a partir de la entrada en vigencia de la segunda etapa de la legislación. Se verificó cuántos productos eximidos de la aplicación de advertencias por la legislación contienen exceso de azúcar, sodio o grasas saturadas según los criterios de la Organización Panamericana de la Salud (OPS).

**Resultados.:**

El 76,1% de los productos superaba al menos uno de los umbrales para el azúcar, el sodio o las grasas saturadas vigentes a partir de septiembre de 2021. La proporción de productos eximidos de presentar advertencias por la legislación y que contienen exceso de azúcar, sodio o grasas saturadas según la OPS será 4,2, 3,4 y 2,3 veces menor, respectivamente, a partir de la segunda etapa.

**Conclusión.:**

Durante la primera etapa de vigencia de la ley, 58%, 42% y 28,2% de los productos eximidos de la presentación de advertencias contenían exceso de azúcar, grasas saturadas o sodio, respectivamente, según la OPS.

## Abstract (en)

**Objective.:**

Identify the nutritional profile of food products exempted from presenting one or more front-ofpackage nutritional warnings during the first stage of Law 30,021 on the Promotion of Healthy Eating for Children and Adolescents in Peru.

**Methods.:**

Data were collected on 188 products from points of sale in the city of Lima. The convenience sample included products that until September 17, 2021 were exempted by the legislation from presenting any of the warning labels for sugar, sodium, or saturated fats. An assessment was made of the proportion of products that would be required to apply one or more warning labels when the second stage of the legislation takes effect. It was also calculated how many products exempted from the application of warning labels contain excess sugar, sodium, or saturated fats, according to the criteria of the Pan American Health Organization (PAHO).

**Results.:**

76.1% of the products exceeded at least one of the thresholds for sugar, sodium, or saturated fats that entered in force in September 2021. The proportion of products that the law exempts from warnings and that contain excess sugar, sodium, or saturated fats according to PAHO will be 4.2, 3.4, and 2.3 times lower, respectively, in the second stage.

**Conclusion:**

During the first stage of the law’s entry into force, 58%, 42%, and 28.2% of the products exempted from the presentation of warning labels contained excess sugar, saturated fats, or sodium, respectively, according to PAHO.

## RESUMO

**Objetivo.:**

Identificar o perfil nutricional de produtos alimentícios isentos da aplicação de um ou mais selos de advertência nutricional na parte frontal da embalagem durante a primeira etapa de vigência da Lei 30021 de Promoção da Alimentação Saudável para Crianças e Adolescentes do Peru.

**Métodos.:**

Foram coletados dados de 188 produtos em pontos de venda da cidade de Lima. A amostra por conveniência incluiu produtos que, até 17 de setembro de 2021, estavam isentos, pela legislação, de apresentar quaisquer advertências para açúcar, sódio ou gorduras saturadas. Foi avaliada a proporção de produtos que seriam obrigados a apresentar um ou mais selos de advertência quando a segunda etapa da legislação entrasse em vigor. Verificou-se quantos produtos isentos da apresentação de advertências, pela legislação, contêm excesso de açúcar, sódio ou gorduras saturadas, segundo os critérios da Organização Pan-Americana da Saúde (OPAS).

**Resultados.:**

76,1% dos produtos ultrapassaram pelo menos um dos limiares para açúcar, sódio ou gorduras saturadas vigentes a partir de setembro de 2021. A proporção de produtos isentos de apresentar advertências, pela legislação, e que contêm excesso de açúcar, sódio ou gorduras saturadas, segundo a OPAS, será 4,2, 3,4 e 2,3 vezes menor, respectivamente, a partir da segunda etapa.

**Conclusão.:**

Durante a primeira etapa de vigência da lei, 58%, 42% e 28,2% dos produtos isentos da apresentação de advertências continham excesso de açúcar, gorduras saturadas ou sódio, respectivamente, segundo a OPAS.

Con el aumento del poder de compra de la población en el Perú, los cambios socioculturales ocurridos y la expansión de las actividades políticas y comerciales de corporaciones fabricantes de productos alimentarios procesados y ultraprocesados, el problema del sobrepeso y la obesidad en el Perú se ha incrementado de forma exponencial, tanto en adultos como en niños, principalmente en zonas urbanas (1-4). Según el Informe del Estado Nutricional en el Perú en 2009-2010, entre los niños de 5 a 9 años la prevalencia de sobrepeso era de 15,5% y la de obesidad de 8,9% (5). Esta situación empeoró en los años siguientes, y en 2013-2014 la prevalencia de sobrepeso se elevó a 17,5% y la de obesidad a 14,8% (6). En un informe más reciente sobre el estado nutricional de mujeres gestantes y niñas y niños menores de cinco años que acceden a establecimientos de salud del Ministerio de Salud se reportó que el sobrepeso y la obesidad continuaron aumentando hasta el año 2020 (7).

Los altos índices de sobrepeso y obesidad de la población peruana, en especial de niños, niñas y adolescentes, impulsaron al Congreso de la República a aprobar en 2013 la Ley de Promoción de la Alimentación Saludable para Niños, Niñas y Adolescentes Nº 30021 (8) que establece, entre otros aspectos, un sistema de advertencias publicitarias para productos alimentarios envasados que contengan grasas trans o que superen determinados niveles de azúcar, sodio y grasas saturadas. En ella se dispuso que los parámetros deberían basarse en el conjunto de recomendaciones de la Organización Panamericana de la Salud/Organización Mundial de la Salud (OPS/OMS) (8).

Con la finalidad de poner en marcha la Ley 30021, el Ministerio de Salud aprobó mediante Decreto Supremo 017-2017-SA (9) los parámetros para definir cuándo un producto tiene un contenido alto de azúcar, sodio o grasas saturadas y debe llevar la advertencia correspondiente. Con relación a las grasas trans se definió que, cualquiera sea su contenido, el producto debería llevar la advertencia correspondiente.

La aplicación de los parámetros se estableció en dos etapas; la primera entró en vigencia el 16 de junio de 2019 y la segunda a partir del mes 39 de la aprobación del Manual de Advertencias (10), es decir, el 17 de septiembre de 2021, fecha a partir de la cual rigen los nuevos parámetros que definen qué productos deben llevar una advertencia, más estrictos que los primeros.

Las advertencias publicitarias constituyen un elemento de información que permite a los consumidores identificar rápidamente un producto que contiene grasas trans o exceso de azúcar, sodio o grasas saturadas a fin tomar decisiones informadas. Las advertencias consisten en octógonos con fondo de color negro y contorno blanco, y texto en letras blancas. Van acompañadas de una leyenda con fondo blanco y letras negras que expresa “evitar su consumo excesivo” para productos con alto contenido de azúcar, grasas saturadas o sodio, o una leyenda que dice “evitar su consumo”, para productos con grasas trans.

Es importante mencionar que, al contrario de lo que indicaba la ley, el Decreto Supremo 017-2017-SA que finalmente se aprobó no adoptó las recomendaciones de la OPS/OMS. Los umbrales adoptados tanto en la primera como en la segunda etapa de la legislación peruana para definir cuándo un producto contiene exceso de azúcar, grasas saturadas o sodio difieren de los estipulados por el Modelo de Perfil de Nutrientes de la OPS (11). En consecuencia, es posible que con la entrada en vigencia de los nuevos puntos de corte productos con exceso de azúcar, sodio o grasas saturadas sigan eximidos de presentar la advertencia.

Por esas razones, el objetivo de este estudio fue identificar el perfil nutricional de una muestra de productos alimentarios procesados y envasados eximidos de presentar alguno de los sellos de advertencia “Alto en” azúcar, sodio o grasas saturadas en el frente del envase durante la primera etapa de vigencia de la Ley peruana 30021 de Promoción de la Alimentación Saludable para Niños, Niñas y Adolescentes y su reglamento.

## MATERIALES Y MÉTODOS

Se diseñó un estudio de caso transversal con una muestra no probabilística seleccionada por conveniencia, en la cual se analizaron 188 productos recogidos al azar en diversos supermercados y bodegas en la ciudad de Lima durante los meses de agosto a diciembre de 2020. El único criterio de inclusión de los productos en la muestra es que estuvieran eximidos para la primera etapa de implementación de la Ley Nº 30021 de la colocación de una o más advertencias para azúcar, sodio o grasas saturadas por contener menos azúcar, grasas saturadas o sodio que lo estipulado por los parámetros de la primera etapa. Se seleccionaron productos nacionales (n=166) e importados (n=22) fabricados por diferentes empresas (61 empresas distintas) y pertenecentes a distintas categorías, como galletas saladas y dulces; productos de panificación (panes de molde, tortillas, panetón, bizcochuelos y otros); gaseosas y bebidas saborizadas; helados: yogures; aperitivos a base de papas, leguminosas y cereales; carnes procesadas; cereales para desayuno; salsas y aderezos; y productos para untar.

Se tomaron fotografías y se analizó la información nutricional consignada en cada una de las etiquetas de los productos a fin de determinar si les correspondería colocar alguna advertencia a partir de la segunda etapa de la aplicación de la ley (5,9,10).

Los productos sólidos que deberían llevar una advertencia “Alto en azúcar”, “Alto en sodio” o “Alto en grasas saturadas” antes del 17 de septiembre de 2021 son aquellos cuyas cantidades de azúcar total, sodio y grasas saturadas sean mayores o iguales a 22,5 g/100 g, 800 mg/100 g y 6 g/100 g de producto, respectivamente. En el caso de los productos líquidos se aplicaban los siguientes límites: 6 g de azúcar total/100 ml, 100 mg de sodio/100 ml y 3 g de grasas saturadas/100 ml de producto. En la segunda etapa, con inicio el 17 de septiembre de 2021, los límites pasan a 10 g de azúcar total/100 g, 400 mg de sodio/100 g y 4 g de grasas saturadas/100 g de producto sólido, y a 5 g de azúcar total/100ml en productos líquidos. Los límites para el sodio y las grasas saturadas en productos líquidos no sufren alteración entre la primera y la segunda etapa. Se estimó la proporción de productos de la muestra que pasarían a ser clasificados con un perfil nutricional “Alto en” azúcar, sodio o grasas saturadas según los umbrales de la segunda etapa de la legislación y que, consecuentemente, estarán obligados a aplicar sellos de advertencia (5,9,10).

Finalmente, se estimó la proporción de productos que tienen un perfil nutricional clasificado como excesivo en azúcares, grasas saturadas o sodio según la OPS, usando el criterio establecido en el Modelo de Perfil de Nutrientes de la OPS (11).

## RESULTADOS

De los 188 productos analizados, que al momento de la recolección de datos no estaban obligados a presentar una o más advertencias nutricionales para alguno de los componentes críticos analizados (azúcar, sodio o grasas saturadas), 152 eran sólidos (contenido expresado en gramos o kilogramos) y 36 eran líquidos (contenido expresado en mililitros o litros).

Al verificar el contenido nutricional declarado por los fabricantes en sus etiquetas y compararlo con los nuevos parámetros en vigencia a partir del 17 de septiembre de 2021 (segunda etapa) se encontró que, de los 188 productos 143 (123 sólidos y 20 líquidos) deberán llevar al menos una advertencia (octógono), lo cual representa el 76,1% de la muestra analizada. En el cuadro 1 se detalla la proporción de productos que se espera encontrar con nuevas advertencias aplicadas desagregadas por cantidad y tipo de advertencias para el total y para los grupos de productos sólidos y líquidos.

**CUADRO 1.** Número y porcentaje de productos de la muestra que pasarían a estar obligados a presentar una o más advertencias nutricionales con la entrada en vigor de los parámetros de la segunda etapa de implementación de la Ley peruana 30021 de Promoción de la Alimentación Saludable para Niños, Niñas y Adolescentes

Cantidad y tipos de advertencias que pasarían a figurar en el frente del envase de productos alimentarios	Total (n=188)		Sólidos (n=152)		Líquidos (n=36)	
	n	%	n	%	n	%
Una o más advertencias	143	76,1%	123	80,9%	20	55,6%
Una nueva advertencia	71	37,8%	51	33,6%	20	55,6%
sodio	20	10,6%	20	13,2%	0	0%
azúcar	50	26,6%	30	19,7%	20	55,6%
grasas saturadas	1	0,5%	1	0,7%	0	0%
Dos nuevas advertencias	61	32,4%	61	40,1%	0	0%
sodio y azúcar	7	3,7%	7	4,6%	0	0%
sodio y grasas saturadas	28	14,9%	28	18,4%	0	0%
azúcar y grasas saturadas	26	13,8%	26	17,1%	0	0%
Tres nuevas advertencias (sodio, azúcar y grasas saturadas)	11	5,9%	11	7,2%	0	0%

***Fuente:*** Elaboración propia a partir de los resultados presentados.

**FIGURA 1.**
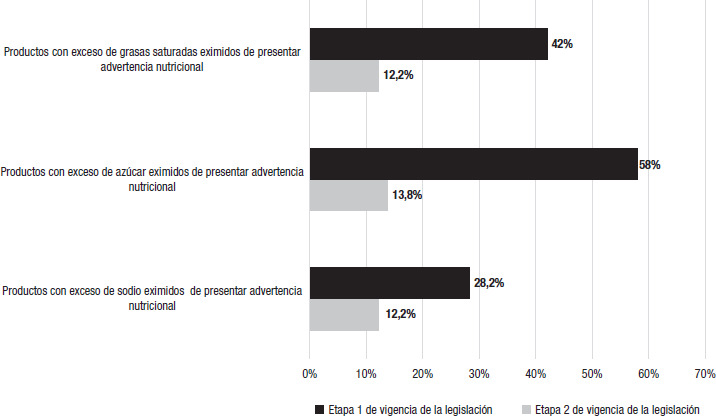
Proporción de productos con exceso de azúcar, sodio o grasas saturadas según los criterios de la OPS que están eximidos de presentar advertencias nutricionales en el frente del envase según la legislación peruana (n=188)

***Fuente*:** Elaboración propia a partir de los resultados presentados.

Seis productos que son para beber se venden por peso (expresado en gramos o kilogramos) y de esta manera se acogen a los parámetros de los productos sólidos, mucho más tolerantes que los dirigidos a los líquidos, y en consecuencia no presentaban advertencias.

Se verificó que más del 50% de los productos contenían exceso de azúcar (n=109), el 42% de los productos contenían exceso de sodio (n=79), y más del 25% de los productos contenían exceso de grasas saturadas (n=53) según los criterios de la OPS. No obstante, estaban eximidos de presentar advertencia sobre estos nutrientes de acuerdo con la primera etapa de vigencia de la legislación (figura 1). Con la entrada en vigencia de la segunda etapa de la legislación, todavía habrá productos con exceso de esos nutrientes, que estarán eximidos de la aplicación de las respectivas advertencias (figura 1).

## DISCUSIÓN

Los resultados del análisis de la información nutricional disponible en el etiquetado de los productos indican que la primera etapa de vigencia de la legislación sobre el etiquetado de productos alimentarios en Perú exime de la aplicación de advertencias en el frente del envase a una gran propoción de productos que contienen exceso de azúcar, sodio y grasas saturadas, según los criterios de la OPS. La entrada en vigor de la segunda etapa de la legislación reducirá sustancialmente la proporción de productos con exceso de azúcar, sodio o grasas saturadas que estarán eximidos de presentar advertencias.

El Decreto Supremo Nº 017-2017-SA (9) y el Manual de Advertencias aprobado por el Decreto Supremo Nº 012-2018-SA (10) introdujeron diversas disposiciones que han alterado las normas contenidas en la Ley N° 30021 (5). Entre ellas, los umbrales establecidos para definir los productos que deben aplicar advertencias en el frente del envase no coinciden con la recomendación de la OPS (11), lo que permite que un gran número de productos con exceso de azúcar, sodio y grasas saturadas sean comercializados sin brindar al consumidor información que alerte sobre el exceso de estos nutrientes en el producto.

**FIGURA 2.**
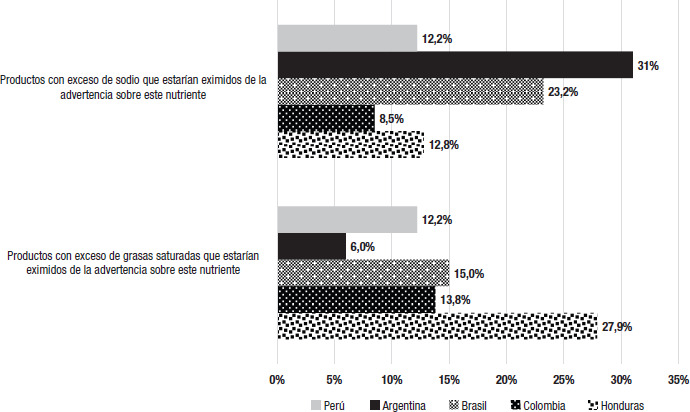
Proporción de productos comercializados en países de América Latina con exceso de grasas saturadas o sodio según la OPS, que estarían eximidos de la presentación de advertencias nutricionales de acuerdo con los umbrales de la segunda etapa de la legislación peruana

Este es el primer estudio que estima la proporción de productos con exceso de azúcar, sodio o grasas saturadas según los criterios de la OPS que están eximidos de presentar la advertencia por los umbrales de una legislación nacional. Sin embargo, otros estudios permiten comparar la proporción de productos clasificados como excesivos en algunos de esos nutrientes utilizando los umbrales adoptados en la tercera etapa de vigencia de la legislación chilena, que coinciden con los umbrales de la segunda etapa adoptada en Perú y los criterios definidos por la OPS (11). La proporción de productos con exceso en nutrientes críticos según la OPS que no llevarían una advertencia de acuerdo con los umbrales de Chile (tercera etapa) o Perú (segunda etapa), para grasas saturadas es un poco mayor en Brasil (15%) y en Colombia (13,8%), más que el doble en Honduras (27,9%) y la mitad en Argentina (6%), y para sodio es casi el triple en Argentina (31%), casi el doble en Brasil (23,2%), similar en Honduras (12,8%) y menor en Colombia (8,5%), con relación a lo reportado en el presente estudio (12-15) (figura 2).

***Fuente*:** Elaboración propia a partir de los resultados presentados y de los resultados publicados por Duran et al. (12), Mora-Plazas et al. (13), Tricornia et al. (14) y Santana et al. (15).

Es digno de mención que la verificación del cumplimiento de la legislación en base sólo al análisis del etiquetado de los productos se ve dificultada por la ausencia de exigencias legales en cuanto a la presentación de información nutricional. En el Perú la información nutricional en las etiquetas no es obligatoria, salvo en casos específicos. Por esa razón, en el mercado hay muchos productos procesados que omiten la información nutricional, lo que dificulta la verificación por este método, menos costoso que la verificación en laboratorio.

Esta ausencia de obligación y estandarización de la información nutricional permite que cada fabricante declare la información como quiera, o simplemente la omita. Por ejemplo, con relación a la cantidad, algunos productos hacen referencia a su propia porción, lo que requiere usar una calculadora para comparar los contenidos entre productos de su categoría. Otros productos, en cambio, expresan el contenido de nutrientes cada 100 gramos o mililitros de producto, y otros lo declaran por porción y por cada 100 gramos o mililitros. Algunas etiquetas hacen referencia a “grasas totales” y otras a “energía de grasa”, y distintos productos se refieren a calorías, energías y o kilocalorías. Respecto de los azúcares existe una disparidad de términos similar, con referencias a azúcares, carbohidratos, azúcares totales y azúcares añadidas. Todo esto hace muy compleja la información para la población (16-19).

Este estudio presenta algunas limitaciones. El uso de una muestra por conveniencia no permite reportar sobre todo el universo de productos exentos de la aplicación de advertencias de acuerdo con los umbrales establecidos por la legislación peruana. Sin embargo, la muestra estudiada incluye productos pertenecientes a una gran diversidad de categorías y empresas, lo que evita mayores sesgos que podrían resultar de la presencia desequilibrada de una u otra categoría o marca específica de productos. Otra limitación es que el estudio analizó el perfil de nutrientes de los productos con base en la información nutricional brindada a los consumidores en el etiquetado de los productos. El estudio asumió que esa información es correcta y cumple con la legislación que exige la comprobación de análisis laboratorial sobre la composición del producto para registrarlo y autorizar su comercialización en el país (20).

En conclusión, más de la mitad de los productos analizados sin advertencia para el azúcar, dos quintas partes de los productos sin advertencia para el sodio, y más de una cuarta parte de los productos sin advertencia para las grasas saturadas (todos ellos eximidos de presentar el sello de advertencia por no superar los umbrales de la primera etapa de vigencia de la legislación peruana) contenían una cantidad excesiva de azúcar, sodio o grasas saturadas según los límites establecidos para lograr una alimentación saludable de acuerdo con las recomendaciones de la OPS/OMS. Desde la entrada en vigencia de la primera etapa de la legislación peruana, la población encuentra disponible en el mercado productos que pueden comprometer su alimentación y salud por la cantidad excesiva de estos nutrientes, sin contar con una advertencia que comunique de manera eficaz, clara y veraz esa información. La entrada en vigencia de la segunda etapa de la legislación con umbrales más rigurosos de acuerdo con lo establecido en los parámetros técnicos del Decreto Supremo Nº 012-2018-SA permitirá reducir la proporción de productos con exceso de azúcar, sodio o grasas saturadas eximidos de presentar la advertencia, como se ha demostrado para la muestra de productos analizada en este estudio. Como resultado, se espera que el aumento en la proporción de productos que entregan información correcta, veraz y de fácil entendimiento a la población contribuya a mejorar la protección de la alimentación saludable y de la salud de la población en el Perú. Finalmente, esa mejoría esperada podrá alcanzar estándares óptimos cuando todos los productos con exceso de azúcares, grasas saturadas y sodio según la OPS estén obligados por la legislación a colocar las advertencias correspondientes.

## Declaración.

Las opiniones expresadas en este manuscrito son únicamente responsabilidad de los autores y no reflejan necesariamente los criterios ni la política de la *RPSP/PAJPH* o de la Organización Panamericana de la Salud.
